# Scaling Down to Scale Up: A Health Economic Analysis of Integrating Point-of-Care Syphilis Testing into Antenatal Care in Zambia during Pilot and National Rollout Implementation

**DOI:** 10.1371/journal.pone.0125675

**Published:** 2015-05-13

**Authors:** Katharine D. Shelley, Éimhín M. Ansbro, Alexander Tshaka Ncube, Sedona Sweeney, Colette Fleischer, Grace Tembo Mumba, Michelle M. Gill, Susan Strasser, Rosanna W. Peeling, Fern Terris-Prestholt

**Affiliations:** 1 Department of Epidemiology & Biostatistics, George Washington University School of Public Health, Washington, DC, United States of America; 2 Department of Clinical Research, London School of Hygiene and Tropical Medicine, London, United Kingdom; 3 Elizabeth Glaser Pediatric AIDS Foundation, Lusaka, Zambia; 4 Department of Global Health and Development, London School of Hygiene and Tropical Medicine, London, United Kingdom; 5 HIV/AIDS STI Programme, Ministry of Health, Lusaka, Zambia; 6 Elizabeth Glaser Pediatric AIDS Foundation, Washington, DC, United States of America; Johns Hopkins University, UNITED STATES

## Abstract

Maternal syphilis results in an estimated 500,000 stillbirths and neonatal deaths annually in Sub-Saharan Africa. Despite the existence of national guidelines for antenatal syphilis screening, syphilis testing is often limited by inadequate laboratory and staff services. Recent availability of inexpensive rapid point-of-care syphilis tests (RST) can improve access to antenatal syphilis screening. A 2010 pilot in Zambia explored the feasibility of integrating RST within prevention of mother-to-child-transmission of HIV services. Following successful demonstration, the Zambian Ministry of Health adopted RSTs into national policy in 2011. Cost data from the pilot and 2012 preliminary national rollout were extracted from project records, antenatal registers, clinic staff interviews, and facility observations, with the aim of assessing the cost and quality implications of scaling up a successful pilot into a national rollout. Start-up, capital, and recurrent cost inputs were collected, including costs of extensive supervision and quality monitoring during the pilot. Costs were analysed from a provider’s perspective, incremental to existing antenatal services. Total and unit costs were calculated and a multivariate sensitivity analysis was performed. Our accompanying qualitative study by Ansbro et al. (2015) elucidated quality assurance and supervisory system challenges experienced during rollout, which helped explain key cost drivers. The average unit cost per woman screened during rollout ($11.16) was more than triple the pilot unit cost ($3.19). While quality assurance costs were much lower during rollout, the increased unit costs can be attributed to several factors, including higher RST prices and lower RST coverage during rollout, which reduced economies of scale. Pilot and rollout cost drivers differed due to implementation decisions related to training, supervision, and quality assurance. This study explored the cost of integrating RST into antenatal care in pilot and national rollout settings, and highlighted important differences in costs that may be observed when moving from pilot to scale-up.

## Background

Syphilis is a sexually transmitted infection responsible for significant adult and perinatal mortality and morbidity, particularly in low income countries where the majority of the 12 million new cases occur each year [1]. Syphilis prevalence among pregnant women varies across countries in Sub-Saharan Africa (SSA), ranging from 0.1% in Benin up to 14.9% in Zambia [2]. A recent analysis of multinational antenatal surveillance data produced an approximate global estimate of 520,000 adverse outcomes due to maternal syphilis annually, including roughly 212,000 stillbirths or early foetal deaths, 92,000 neonatal deaths, 65,000 preterm or low birth weight infants, and over 151,000 syphilis-infected newborns [3]. Treatment guidelines recommend one to three doses of Benzathine penicillin (BP) in syphilis-positive pregnant women, depending on their stage of infection[4–6]. However, in resource-limited settings, testing and treatment of pregnant women with a single dose of BP has been shown to significantly reduce adverse pregnancy outcomes associated with syphilis [7]. Indeed, testing pregnant women for syphilis is part of the World Health Organization’s (WHO) recommended package of antenatal care (ANC) and has been integrated into ANC policies of most countries in SSA [8,9]. Despite the enormous health burden associated with maternal syphilis, antenatal syphilis testing is not routine in many countries in SSA, with coverage ranging from 0 to 100%. In Zambia, surveillance data from 2012 indicated 27.6% of pregnant women were tested for syphilis during ANC [2].

Until recently, the WHO-recommended syphilis screening tool was the Rapid Plasma Reagin (RPR) test. RPR requires laboratory capacity, electricity, refrigeration and specialist training, which has proved a barrier to consistent availability and accuracy of syphilis testing [9,10]. Research led by WHO/TDR Sexually Transmitted Diseases Diagnostics Initiative validated new point-of-care (POC) rapid syphilis tests (RST) in laboratory and in field settings [11,12]. POC tests have been defined as cheap, simple, hand-held tools which do not require extensive training or refrigeration, are performed near the patient or facility and deliver a rapid result [13,14]. These characteristics lend themselves to mass production, ease-of-use by minimally-trained healthcare workers (HCWs) and implementation in remote settings poorly served by electricity, supply and laboratory networks, thus greatly increasing access to diagnosis.

However, the intrinsic simplicity of POC tests poses challenges for quality maintenance. It is increasingly recognized that successful POC test implementation requires procurement of high quality test kits with good positive predictive value, effective training, quality monitoring systems and regular supervision of testers [15–18], all of which add to implementation costs. Failure to consider these programme aspects can negatively impact on cost-effectiveness of POC tests through over-diagnosis and treatment; yet, few studies assessing these programmatic costs are available and commentators note the unit cost of POC tests is repeatedly underestimated as economists fail to include the costs of implementation, training, human resources and maintenance in their estimates [15].

The cost-effectiveness of testing and treatment of maternal syphilis has been clearly demonstrated [19] and numerous cost models have highlighted that syphilis screening with POC tests is highly cost-effective at WHO-defined thresholds [20,21]. Furthermore, the cost-effectiveness of RSTs was shown to be comparable with RPR in ANC settings in several studies [22,23]. Recent studies demonstrated the feasibility of RST implementation in programmatic settings and highlighted the cost and health system benefits of integrating syphilis with HIV testing within ANC [24–26]. Evidence on the incremental increased costs associated with including a comprehensive quality assurance (QA) system for RST is limited. A recent study in Tanzania found QA costs ranged from $513 to $554 per clinic for a 9-month cost period; the high inter-clinic variability in QA costs was attributed to differences in staff salaries and the transportation costs to reach remote facilities [23].

To our knowledge, no published studies have yet compared the costs of implementing RST in research settings to those of a national scale-up. The primary aim of this study was to analyse the implementation costs of Zambia’s pilot programme, which explored feasibility of RST integration into ANC services, and secondarily, to present preliminary costs observed during Zambia’s national rollout of RST. We explored the key drivers of differences in cost between the two implementation stages, and examined how cost containment measures may have impacted on the quality of syphilis testing delivered to rollout ANC attendees.

## Materials and Methods

### Pilot Study Setting

From 2008–2010, the Elizabeth Glaser Pediatric AIDS Foundation (EGPAF) in partnership with the Centre for Infectious Disease Research in Zambia (CIDRZ) conducted a pilot that examined the feasibility and acceptability of introducing RSTs into prevention of mother-to-child-transmission of HIV programmes in ANC clinics in Zambia. A detailed description of the pilot and results is available elsewhere [25]; in brief, RST was introduced within a variety of ANC settings at 15 pilot facilities in two districts, comprising urban and rural locations, high and low ANC volume clinics and high and low syphilis prevalence (approximately 7% in Mongu and 2.5% in Lusaka). A centralized two-day RST training workshop took place before RST was integrated within existing clinic staffing patterns, patient flow, and clinic processes alongside other routine antenatal POC tests (HIV, malaria, and haemoglobin). The pilot utilised SD BIOLINE Syphilis 3.0, a rapid POC syphilis antibody test produced by Standard Diagnostics (Yongin-Si, South Korea), with a manufacturer reported 99.3% sensitivity and 99.5% specificity in serum versus the gold standard *Treponema pallidum* haemagglutination (TPHA) test [27]; of note, a recent meta-analysis of the diagnostic accuracy of SD Bioline 3.0 in field conditions reported a lower pooled sensitivity (87.9% serum; 83.8% whole blood) and specificity (96.0% serum; 98.4% whole blood)[28]. Pilot-specific QA and quality control (QC) measures were established to ensure high standards of quality management [25,29]. The pilot results showed increased syphilis testing among ANC attendees (79.9% versus 95.6%, p<0.0001) and treatment of syphilis positive pregnant women (51.1% versus 95.2%, p<0.0001), and demonstrated the feasibility of integrating RST within busy urban and rural ANC settings in Zambia [25].

### National Rollout Setting

Following the successful pilot, Zambia adopted RST into national policy and recommended the use of rapid syphilis tests to offer same-day testing and treatment [30,31]. In March 2012, the Zambian Ministry of Health (MOH) launched the first phase of RST rollout in four underserved districts with high rates of maternal mortality: Mansa (Luapula Province), Kalomo (Southern Province), and Lundazi and Nyimba (Eastern Province). There were several key implementation differences between the pilot and rollout with regard to training, supervision, quality management mechanisms, and testing and treatment algorithms. Firstly, both pilot and rollout utilised a cascaded training approach whereby at least one staff member from each participating facility attended a district-level training workshop and, in-turn, trained facility colleagues in RST testing procedure. However, in practice, rollout HCWs received substantially less on-the-job supervision and guidance following initial training. Second, the rollout supervision process involved a 10-day MOH/EGPAF joint visit to rollout districts during May-July 2012, with unannounced visits to selected facilities; whereas, the pilot supervision process involved monthly EGPAF/CIDRZ monitoring visits at all pilot sites for data collection, quality checks and remedial on-site training for poor performers identified through proficiency testing. Third, the QA/QC mechanisms were centrally led during the pilot, with study staff providing materials and feedback; for the rollout, training was provided to district-level laboratory staff in order to assume this role. Fourth, at all pilot sites, treatment was initiated on the basis of a positive RST. RST detects antibodies specific to the causative bacterium *Treponema pallidum*, and does not distinguish between active and past, treated infection; whereas, the rollout introduced different testing algorithms utilising either RST alone, or RST as a screening test followed by RPR as a confirmatory test where this was available. The differences in treatment algorithm are described below. Our interpretation of differentials in the cost data and of the influence of implementation decisions on costs draws heavily on the qualitative findings reported in our accompanying paper by Ansbro et al. (2015, in review), which includes a detailed comparison of implementation differences in scale up from successful pilot to national rollout [32].

### Costing Methods

#### Data collection

Our cost methodology combined an ingredients-based approach, whereby a unit cost is multiplied by a resource quantity to generate a total cost, with a step-down cost accounting approach for facility level data in that joint costs are allocated to activities through cost centres [33,34]. A variety of sources were used for data on inputs, outputs and costs, including: inspection of facility records and registers, clinic observation, expert interviews with clinic and project staff, and review of project accounts. An Excel-based cost collection tool was used to collect the cost information during the pilot and rollout phases [34]. Costs were collected between March and July 2010 from five of the fifteen pilot facilities, including two urban health centres (UHC) in Lusaka plus one UHC and two rural health centres (RHC) in Mongu; facilities were purposively sampled to represent variation in facility size, target population, and location. For the national rollout, costs for the period March to July 2012 were collected from five facilities, including one UHC and three RHCs in Mansa District and one district hospital (DH) in Kalomo; facilities were sampled by convenience from among facilities visited by the MOH supervisory team during July and August 2012. Prevalence data from the urban facility (UHC4) diverged significantly from the average and was considered an outlier. We excluded UHC4 from the cost analysis, but the unique challenges experienced by this facility are discussed in the qualitative paper [32]. Table 1 illustrates key differences across the pilot and rollout facilities.

**Table 1:** Comparison of key characteristics of pilot versus rollout facilities.

	PILOT					ROLLOUT			
Facility Type/Code	UHC1	UHC2	UHC3	RHC1	RHC2	DH1	RHC3	RHC4	RHC5
Province	Lusaka	Lusaka	Western	Western	Western	Southern	Luapula	Luapula	Luapula
District	Lusaka	Lusaka	Mongu	Mongu	Mongu	Kalomo	Mansa	Mansa	Mansa
**General**									
Distance from DHO (KM)	9	10	2	18	76	2	89	42	10
Laboratory on-site	Yes	Yes	Yes	Yes	No	Yes	Yes	No	No
Finger prick or Venepuncture	Both	FP	VNP	FP	FP	Both	Both	FP	Both
RST testing days/week	5	5	1	1	1	1	1	2	1
**Supervision**									
District Supervisory Visits	5	5	5	5	5	—	—	—	—
Program Supervisory Visits	20	20	3	3	3	1	1	1	1
**Quality Assurance/Control**									
External Quality Control	Weekly using pre-manufactured control samples					Inconsistently conducted; only 1 clinic used own positive and negative samples			
External Quality Assurance	Twice during cost period using dried tube specimens					In 1 district, proficiency testing attempted, but DTS results not returned to District Lab			
Confirmatory Retesting	Conducted on 10% of all RST samples					Not included in the national rollout			

DTS = Dry Tube Specimen; FP = Finger Prick; KM = Kilometre; RHC = Rural Health Centre; UHC = Urban Health Centre; VNP = Venepuncture

Both pilot and rollout examined the incremental cost of adding RST screening and treatment onto existing ANC services, i.e. any additional costs to execute syphilis screening and treatment were included but administrative costs to run the health facility were excluded. All research costs to collect data during the pilot and rollout were also excluded. Economic costs were collected retrospectively from the provider’s perspective. Financial and logistical constraints during the rollout evaluation prevented full replication of pilot cost methods, the key difference being that direct observation of rollout RST test performance and clinic flow was not possible, either due to RST stock outs or lack of ANC services scheduled on the day of site visits. Therefore, rollout recurrent staff time estimates are based on pilot costing and interviews with HCWs and experts.

All pilot and rollout costs are presented in 2012 United States Dollars (USD). Pilot costs collected in 2010 Zambian Kwacha (ZKW) were converted to USD using the average exchange rate for 2010 (ZKW 4,743.98 = 1 USD [35]), then adjusted to 2012 USD using a 2-year inflation rate of 5.29% (2010 CPI 218.056 / 2012 CPI 229.594 = 1.0529) from the US Consumer Price Index (CPI) [36,37]. During the rollout, non-supply costs were collected in 2012 ZKW or USD; supply costs were collected in 2011 ZKW and inflation adjusted to 2012 ZKW using Zambia’s CPI (2012 CPI 122.439 / 2011 CPI 115.091 = 1.0638) [38]. All rollout costs in 2012 ZKW were then converted to USD using the average exchange rate for 2012 (ZKW 5,219.83 = 1 USD [39]).

#### Cost Categorisation

Start-up, capital and recurrent cost inputs were collected during pilot and rollout phases. Recurrent and capital costs were further subdivided into facility and central level categories to highlight the implementation costs of incorporating an extensive supervision and QA system during the pilot phase. Start-up is considered an input, rather than an activity, and therefore includes all resources required for training (e.g. personnel, per diems, conference hire, training equipment and supplies, and vehicle transport). For the pilot period only, the start-up also included costs for two district events to launch the RST pilot activities. All start-up costs during the pilot and rollout were annualised over an estimated project life of three years.

Capital costs are generally considered to have a life span of more than one year and cost greater than $100 USD per unit [40]. We included capital cost inputs for vehicles and computers. Annual financial costs were estimated using straightline depreciation, while economic costs were annualised with a 3% discount rate [41]. RPR-related equipment was not included, as this study focussed on incremental costs to existing programs. None of the pilot or rollout sites had an on-site vehicle dedicated to ANC services; thus, pilot vehicle costs included travel in the project, MOH, or district health vehicle for routine study monitoring, delivery of supplies and QA visits. During rollout, the only vehicle costs comprised return travel from Lusaka for supervisory monitoring visits. Vehicles were annualized over a five-year period. The economic costs of computers used for electronic medical records at two pilot facilities in Lusaka were annualised over a three-year period with an allocation factor of 20%. During rollout, the economic costs of computers used during RST training were annualised over three years with an allocation factor ranging from 3 to 12% based on the number of participants attending training from each facility.

Recurrent cost inputs comprised all operating costs throughout the project life, including: personnel, supplies, vehicle fuel and maintenance for supervisory visits, QA/QC, and supervision. Table 2 presents differences between pilot and rollout in terms of unit prices and quantity of healthcare resources consumed for testing commodities. Personnel time and some supplies were considered joint “shared” costs, with an allocation factor applied based on researcher time-motion observation during the pilot. Supply use was not directly observed but was modelled from monthly output data plus a 10% adjustment to account for estimated supply wastage. Supplies for testing and treating male partners were included for the pilot. Treatment with a single dose of BP was assumed during the pilot because follow up doses were not generally recorded in registers and could not be verified. Following the pilot and RST policy adoption, national treatment algorithms outlined a single injection of BP following a reactive RST; where RPR confirmation was unavailable, this was followed by two further weekly doses of BP; where RPR confirmation was available, two further BP doses were given if active syphilis infection was confirmed (a description of algorithms is available in Ansbro et al.) [32]. Two of the four rollout facilities (DH1 and RHC3) utilised confirmatory RPR; for these clinics, three doses of BP are assumed in the costing calculations for RPR-confirmed positives. Male partner testing was inconsistent during rollout; therefore, only male partner treatment costs were included. Shared supplies (e.g. biohazard bags, test tubes and needles for blood draw, sharps bins, gloves, cottonwool, and disinfectant) were given a 25% allocation factor to reflect that four blood tests were routinely conducted on ANC patients (HIV, syphilis, haemoglobin, and malaria). Whereas, supplies used only for syphlis testing (e.g. RST test kit, RST job aid, penicillin, water and needle for BP injection), were given an allocation factor of 100%. For facilities that used both venipuncture and finger prick methods of blood collection, we assumed each method was used 50% of the time.

**Table 2:** Comparison of unit price and quantities of testing and treatment consumables used by facilities during pilot and rollout periods (2012 USD).

		PILOT: March-July 2010						ROLLOUT: March-July 2012				
		Unit Price	UHC1	UHC2	UHC3	RHC1	RHC2	Unit Price	DH1	RHC3	RHC4	RHC5
Syphilis testing	Allocation	$2012	Quantity of consumables†					$2012	Quantity of consumables†			
SD Bioline RST Kit (includes shipping)	1.00	0.65	1,927	2,618	470	158	243	1.15	169	113	125	55
Disposable syringe without needle*	0.25	0.03	964	0	470	0	0	0.05	85	57	0	28
Needle for blood draw* ^	0.25	0.01	964	0	470	0	0	0.17	85	57	0	28
Test tube for blood specimen*	0.25	0.15	964	0	470	0	0	0.14	85	57	0	28
Confirmatory RPR Test Kit	100	0.19	0	0	0	0	0	0.31	12	4	0	0
**Syphilis treatment**	** **		** **	** **			** **					
Disposable syringe w/needle (10ml)^	100	0.07	190	235	117	17	11	0.71	12	15	4	8
Benzathine penicillin (2.5 MU 1 dose)	100	0.33	190	235	117	17	11	0.51	12	15	4	8
Water for BP injection (10ml)	100	0.04	190	235	117	17	11	0.04	12	15	4	8
Partner notification slip	100	0.12	179	169	108	22	13	0.11	12	4	4	8
**Both syphilis testing and treatment**	** **											
Gloves	0.25	0.07	1,942	602	233	155	254	0.06	194	133	130	63
Cottonwool (500g pilot; 1g rollout)	0.25	1.37	1	1	1	1	1	0.01	97	67	65	31
Disinfectant Methylated Spirit	0.25	2.64	1	1	1	1	1	2.45	1	1	1	1
Biohazard bag	0.25	0.26	78	78	16	16	16	0.06	20	20	40	20
Sharps bin	0.25	3.33	20	40	16	16	16	-	-	-	-	-

*For facilities that reported both finger prick and venous blood draw methods for RST, we assumed 50% for each collection type

† Includes 10% supply wastage

^Higher cost vacutainer needle used during rollout period

QA/QC was considered a recurrent input that included personnel, supplies, and transporation costs for distributing and collecting known positive and negative samples for testing at the facility level during the pilot phase; a supplementary table is available with further details of the QA/QC cost calculations (S1 Table). During the rollout, a formal QA/QC system was not implemented and supervision activities occurred infrequently; therefore, the only central level costs for supervision included personnel salaries, accommodation, and fuel costs during the supervision trip. Recurent building utilities and waste management were excluded given the incremental costing approach. See the supplementary table of data inputs and assumptions for additional information (S2 Table).

### Project Outputs

All project outputs were retrospectively collected from facility-level ANC patient registers during the five-month cost collection period for both pilot and rollout. Outputs included number of pregnant women attending first ANC visit, number of pregnant women and partners screened for syphilis with RST, number of syphilis-reactive tests, and number of women and partners treated for syphilis. Unit economic costs were calculated per patient tested and per patient treated at each facility, and a facility average was calculated.

### Sensitivity Analysis

We performed univariate and multivariate sensitivity analyses to assess the influence of key inputs on overall unit costs. Cost inputs that were not directly observed, were highly uncertain or differed substantially between the pilot and rollout periods were varied in the sensitivity analysis, including project life years (1 to 5 years), supply wastage rate (0 to 50%), blood collection method (finger prick versus venepuncture), price of RST kits ($0.65 to $1.15), and coverage of RST among first ANC attendees (25% to 100%). Best and worst case scenarios were estimated by applying the minimum and maximum value for all parameters varied in the sensitivity analysis.

### Ethical Review

The RST pilot protocol was approved by the University of Zambia Biomedical Research Ethics Committee (UNZAREC), University of Alabama at Birmingham’s Institutional Review Board, the WHO’s Research Ethics Review Committee, and MOH Zambia. The follow-up research to collect costs during the national RST rollout was approved by LSHTM University Ethics, UNZAREC and by the Permanent Secretary of the Zambian MOH. Additionally, written informed consent to conduct data collection related to national rollout was obtained from each Provincial and District Health Office. During both pilot and rollout, individual patient consent was not administered. De-identified patient outcome data were retrospectively abstracted from routinely collected health register data into aggregate monthly summaries.

## Results and Discussion

During the March-July 2010 pilot study period, 4,949 pregnant women presented for a first ANC visit, of whom 97.3% (n = 4817) were screened for syphilis, 9.3% (n = 447) tested reactive, and of these, 89.3% (n = 399) were treated for maternal syphilis at five facilities in two districts (Table 3). The average prevalence based on reactive RST was notably different between the pilot facilities with a range from 6% to 23%, and a notable difference between urban Lusaka (8.1%) and rural Mongu (17.7%) districts [25]. During the same period in 2012, 1,210 pregnant women attended first ANC at four rollout facilities in two different districts. Of the 34.8% (n = 421) who were screened for syphilis, 6.2% (n = 26) had a reactive result (or tested positive), but facility-level prevalence ranged from 3.5% to 14.0%. Treatment coverage with the first dose of penicillin averaged 84.6%, and ranged from 50 to 100% across rollout sites.

**Table 3:** Comparison of screening and treatment cascade of outputs for pilot and rollout facilities.

	PILOT: March-July 2010						ROLLOUT: March-July 2012					
Facility Type/Code	UHC1	UHC2	UHC3	RHC1	RHC2	Total	DH1	RHC3	RHC4	RHC5	Total	
First ANC visit	1724	2379	484	162	200	4949	638	271	144	157	1210	
Women screened	1707	2348	424	142	196	4817	154	103	114	50	421	
*% screened*	*99*.*0*	*98*.*7*	*87*.*6*	*87*.*7*	*98*.*0*	*97*.*3*	*24*.*1*	*38*.*0*	*79*.*2*	*31*.*8*	*34*.*8*	
Women reactive	163	154	98	20	12	447	11	4	4	7	26	
*% reactive*	*9*.*5*	*6*.*6*	*23*.*1*	*14*.*1*	*6*.*1*	*9*.*3*	*7*.*1*	*3*.*9*	*3*.*5*	*14*.*0*	*6*.*2*	
Women treated	134	154	87	14	10	399	11	4	2	5	22	
*% reactive treated*	*82*.*2*	*100*.*0*	*88*.*8*	*70*.*0*	*83*.*3*	*89*.*3*	*100*.*0*	*100*.*0*	*50*.*0*	*71*.*4*	*84*.*6*	
Partners screened	45	32	3	2	25	107	Not available*					
*% partners screened*	*2*.*64*	*1*.*36*	*0*.*71*	*1*.*41*	*12*.*76*	*2*.*22*						
Partners treated	39	60	19	1	0	119	*NA*	2	2	2	6	
Total screened	1752	2380	427	144	221	4924	154	103	114	50	421	
Total treated	173	214	106	15	10	518	11	6	4	7	28	

*Data on partner testing was inconsistently collected during rollout period.

### Total Costs

**Fig 1:**
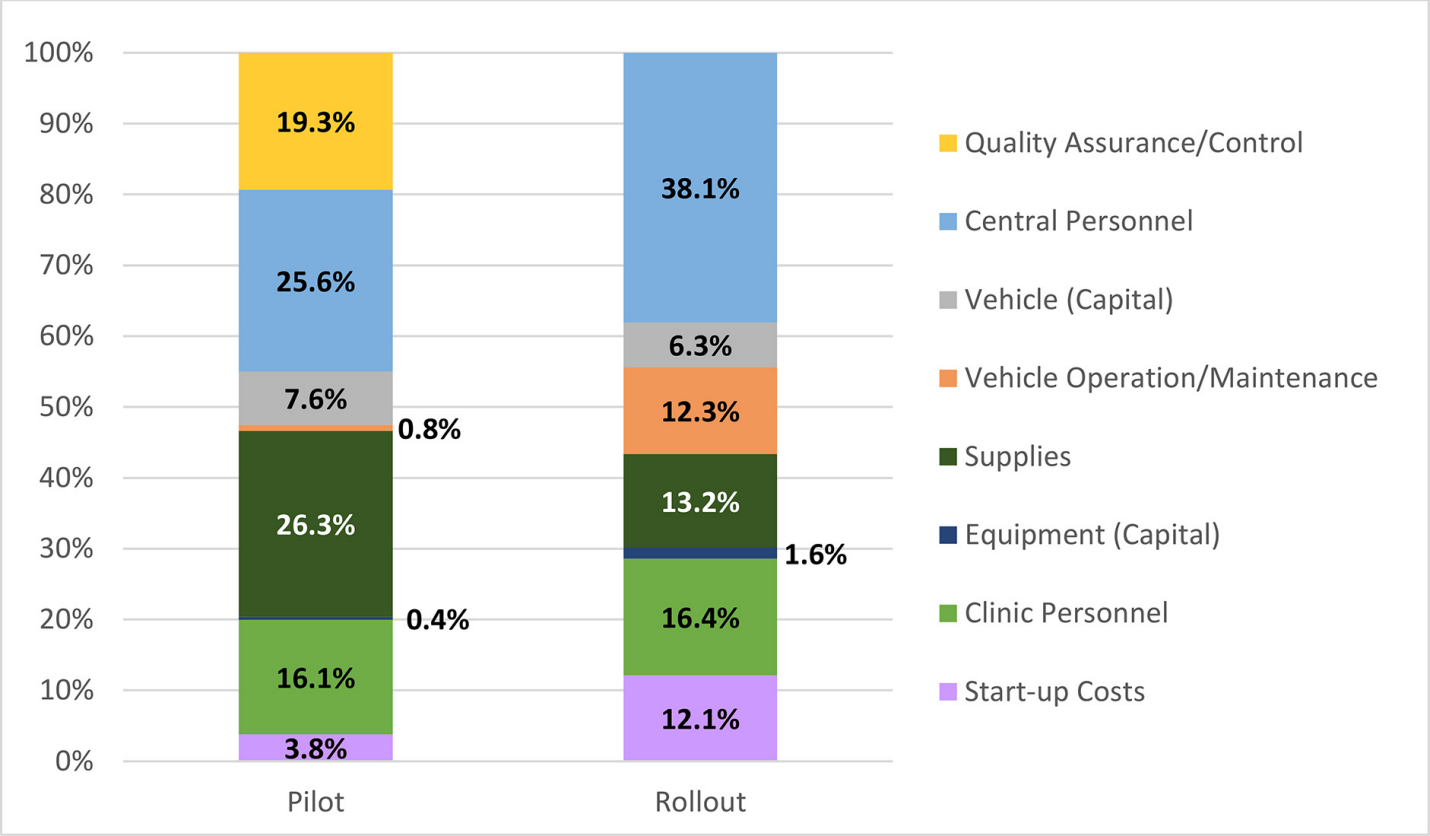
Economic cost drivers at surveyed pilot and rollout facilities. Central-level supervision (including QA/QC costs during pilot) accounted for over half of costs. Supervision, start-up, and health facility costs (supplies, personnel) were also major cost drivers.

Across pilot facilities, total economic costs ranged from $1,952 to $4,799 (Table 4). Central-level costs (supervision and QA/QC) comprised the majority of costs (53.4%), followed by facility-level costs (42.8%) and start-up costs (3.8%). Overall, supervision (34.0%), supplies (26.3%), clinic personnel (16.1%), and QA/QC (19.3%) were the major cost drivers during the pilot (Fig 1). Pilot QA/QC costs ranged from $204 to $883 and supervision costs (central-level personnel and vehicle costs) averaged around $1,500 per Lusaka facility and $770 for Mongu facilities which were visited less frequently. In comparison, during rollout, the total economic costs ranged from $882 to $1,719 (Table 4); health facility costs (31.2%) comprised a lower proportion of total costs compared to the pilot given the far lower RST coverage (Fig 1). During the rollout, supervision by central level personnel (38.1%) and the associated transport costs (18.6%) were major cost drivers. Clinic personnel (16.4%), supplies (13.2%), and start-up (12.1%) were also major cost components (Fig 1). In-depth supervision and QA/QC mechanisms put in place during the pilot phase were not similarly implemented during rollout, yet central-level pilot costs (53.4%) were similar to rollout costs (56.7%), discussed below.

**Table 4:** Economic cost comparison between pilot and rollout facilities.

	PILOT: March-July 2010 (2012 USD)					ROLLOUT: March-July 2012 (2012 USD)			
Facility Type/Code	UHC1	UHC2	UHC3	RHC1	RHC2	DH1	RHC3	RHC4	RHC5
**START-UP COSTS**									
Training	71.64	107.24	89.56	88.71	85.28	334.70	77.44	77.44	80.48
Project Launch	13.85	13.85	42.31	42.31	42.31	-	-	-	-
**Total Start-up Costs**	85.49	121.09	131.86	131.02	127.58	334.70	77.44	77.44	80.48
**HEALTH FACILITY COSTS**									
Clinic Personnel	1,013.45	1,011.39	213.37	66.64	227.00	453.35	119.10	155.20	45.32
Supplies	1,469.92	1,888.47	422.53	143.76	197.11	227.48	160.20	154.15	78.44
Equipment (Capital)	35.99	27.92	-	-	-	42.32	10.26	10.26	10.26
**Total Health Facility Costs**	2,519.36	2,927.78	635.90	210.40	424.11	723.16	289.56	319.61	134.03
**CENTRAL-LEVEL COSTS**									
Supervision	1,504.91	1,516.18	762.52	767.72	784.97	661.37	667.36	667.36	667.36
*Central Personnel*	*1*,*075*.*66*	*1*,*075*.*66*	*622*.*93*	*622*.*92*	*622*.*93*	*493*.*58*	*431*.*77*	*431*.*77*	*431*.*77*
*Vehicle Operation/Maintenance*	*10*.*41*	*21*.*68*	*23*.*76*	*28*.*97*	*46*.*22*	*86*.*56*	*163*.*30*	*163*.*30*	*163*.*30*
*Vehicle (Capital)*	*418*.*84*	*418*.*84*	*115*.*83*	*115*.*83*	*115*.*83*	*81*.*22*	*72*.*29*	*72*.*29*	*72*.*29*
Quality Assurance/Control	203.88	234.20	870.56	842.89	882.69	Not collected*			
*Incoming Inspection*	*2*.*02*	*2*.*02*	*2*.*02*	*2*.*02*	*2*.*02*				
*External Quality Control*	*73*.*02*	*85*.*13*	*236*.*41*	*236*.*54*	*257*.*66*				
*External Quality Assurance*	*114*.*35*	*132*.*57*	*617*.*64*	*589*.*85*	*608*.*52*				
*Confirmatory Retesting*	*14*.*49*	*14*.*49*	*14*.*49*	*14*.*49*	*14*.*49*				
**Total Central Costs**	1,708.78	1,750.38	1,633.08	1,610.61	1,667.66	661.37	667.36	667.36	667.36
**Total Cost for Project Period**	4,313.64	4,799.25	2,400.84	1,952.03	2,219.35	1,719.23	1,034.36	1,064.41	881.86

*A formal QA/QC system was not established during the rollout; informal QA/QC activities were conducted within supervision-monitoring visits.

### Unit Costs

During the pilot, the average unit cost was $3.19 per person screened and $30.34 per person treated, with substantial variation in unit cost across pilot facilities, which ranged from $2.02 to $13.75 per person screened and from $22.43 to $221.94 per person treated (Table 5). In the rollout phase, the average unit cost per woman screened ($11.16) was over triple that of the pilot, ranging from $9.34 to $17.64, and the average cost per woman treated was $167.85, ranging from $125.98 to $266.10. One key difference between implementation phases was the increase in RST kit costs from $0.65 during the pilot to $1.15 during the rollout. Economies of scale were noticeable at the high volume urban facilities of the pilot, where the relatively high fixed costs associated with start-up, ongoing supervision, and QA/QC were spread across a larger ANC patient population. When summarised by urban versus rural health centres, pilot unit costs were $2.53 per person screened versus $11.49, respectively. For the rollout, there was no difference between the average unit cost per person screened ($11.16) at RHCs and the DH1 facility.

**Table 5:** Screening and treatment unit cost comparison between pilot and rollout facilities.

	PILOT: March-July 2010 (2012 USD)					ROLLOUT: March-July 2012 (2012 USD)			
Facility Type/Code	UHC1	UHC2	UHC3	RHC1	RHC2	DH1	RHC3	RHC4	RHC5
**Unit Costs**									
Per person screened	2.46	2.02	5.62	13.75	10.04	11.16	10.04	9.34	17.64
Per person treated	24.93	22.43	22.65	130.14	221.94	156.29	172.39	266.10	125.98
**Average Unit Costs**	**All facilities**					**All facilities**			
Average per person screened	3.19					11.16			
Average per person treated	30.28					167.85			
**Average Unit Costs, Urban vs. Rural**	**UHCs**			**RHCs**		**DH1**	**RHCs**		
Average per person screened	2.53			11.49		11.16	11.16		
Average per person treated	23.35			166.86		156.29	175.33		
**Average Unit Costs, excludes central**	**All facilities**					**All facilities**			
Average per person screened	1.49					4.84			
Average per person treated	14.12					72.73			

### Sensitivity Analysis

**Fig 2:**
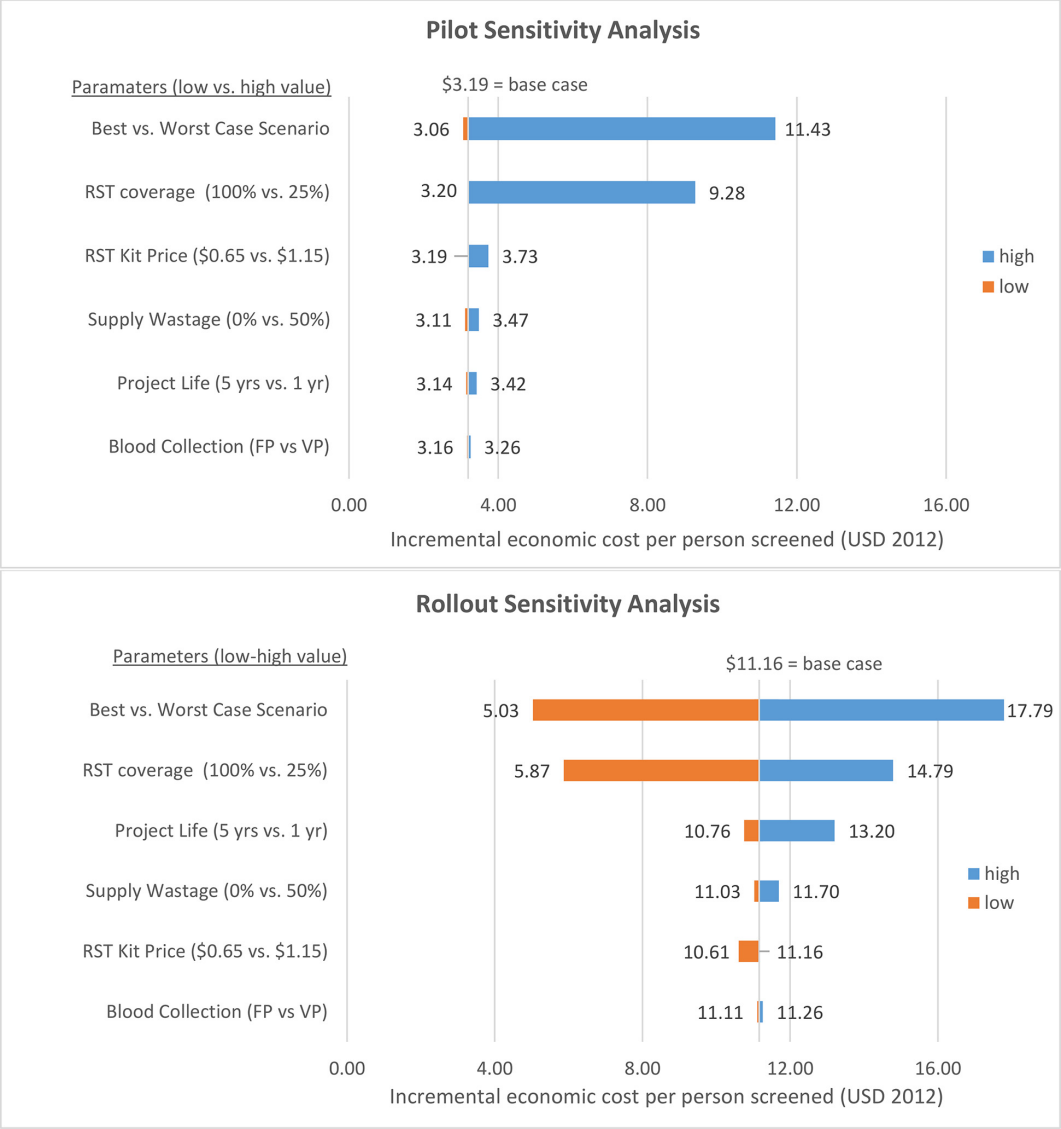
Tornado diagram of one-way and multivariate sensitivity analyses of incremental cost per person screened at pilot and rollout facilities (2012 USD). A range of uncertain parameters was varied in one-way sensitivity analyses; parameters are displayed along the vertical axis. The solid vertical line indicates the base case incremental cost per person screened for syphilis ($3.19 during pilot; $11.16 during rollout). The horizontal bars represent the range of cost per person screened when varying the associated parameter from the low to high values indicated in parentheses. The best versus worst case scenario is a multivariate representation when all parameters are set to the low versus high values. During pilot and rollout, the cost per person screened was most highly sensitive to RST coverage among ANC attendees. FP = Finger prick; VP = Venepuncture.

We noted differences in the impact of varying key parameters on the cost per person screened between pilot and rollout. Results of the univariate sensitivity analysis indicated that unit costs were particularly sensitive to coverage of RST services in both pilot and rollout. For the pilot, decreasing RST coverage from the base case (97%) to 25% resulted in an increase from $3.19 to $9.28 in average unit cost per person screened, which was much closer to the unit cost during the rollout period where testing coverage was only 35%. Similarly, for the rollout, varying RST coverage from the base case (35%) to 100% coverage decreased the cost per person screened from $11.16 to $5.87, again far closer to the pilot unit cost ($3.19) (Fig 2), and more reflective of the unit screening cost of $3.10 (minus QA/QC and supervisory costs) modelled by Larson et al. with RST coverage of 62% [42]. Unit costs were also sensitive to RST kit price and the wastage rate for supplies during both pilot and rollout phases, which reflects the substantial proportion of overall costs dedicated to supplies. During rollout, the unit cost estimate was sensitive to the length of the project life, reflecting the comparatively higher proportion of training start-up costs during rollout (12.1%) versus pilot (3.8%). The multivariate best/worst case scenario analysis suggests that the worst case scenario during the pilot ($11.43) would approximate the average unit cost of the rollout period. The rollout unit cost was much greater, ranging from $5.03 to $17.79 per person screened.

### Cost Differentials in Moving from Pilot to Scale-Up

The average unit cost ($3.19) per person screened in the Zambian pilot study setting is consistent with the range of published data on RST in ANC settings from a variety of low- and middle-income countries, ranging from $1.02 to $6.96 [22,23,43,44]. However, our results highlight the substantial increase in unit cost ($11.16) per person screened observed during Zambia’s preliminary national rollout of RST. This is partially attributable to higher supply costs for RST kits during the rollout ($1.15 versus $0.65 during the pilot) but is primarily driven by far lower testing rates; 97.3% of women were screened at first ANC visit during the pilot, compared to 34.8% during rollout. The low coverage can in part be explained by supply constraints where three of four rollout sites reported repeated RST stock outs, discussed below, but may also be attributable to provider failure to test. For example, DH1 had low testing rates despite no reported RST stock outs (Table 3). The low rate of testing coverage meant far lower volumes of tests were conducted during the rollout cost period (N = 421) compared to pilot cost period (N = 4,817), also reducing economies of scale. Treatment rates were similarly high during pilot and rollout phases (85–90%). The average cost per person treated during rollout was nearly five times ($148) that of the pilot ($30), again driven by the far lower testing coverage during rollout.

Urban and rural cost differentials varied. During the pilot, the average cost per person screened was $2.53 in urban facilities and $11.49 in rural facilities. At urban sites, higher facility-level costs were mitigated by the economies of scale afforded by high patient volumes. Costs to implement QA/QC in facilities in rural districts were elevated due to long travel distances from Lusaka. During the rollout, low RST coverage at DH1 (24%) resulted in an elevated screening unit cost ($11.16), which was unexpected for a high volume ANC setting. The low coverage meant the average screening unit cost at DH1 was comparable to the lower volume, rural-based rollout facilities. A recent pre-post study of RST rollout in 18 ANC facilities in one of the Zambian districts included here, reported baseline antenatal syphilis testing (using RPR) of 10%, which increased to 62% during the 12 months after RST introduction [45]. Thus, testing rates are likely to have improved in the time since our data were collected.

Clinic personnel costs comprised approximately 16% of total costs for both pilot and rollout, while supplies contributed a higher proportion (26%) to costs during pilot versus rollout (13%) which was driven by higher RST coverage during the pilot. There were notable differences in cost drivers between the phases, in part due to the absence of comprehensive supervision and quality monitoring systems during the rollout (Fig 1).

### Monitoring and Supervision Costs

During the pilot, QA/QC accounted for about 20% of costs and general supervision accounted for 34% of costs driven by transport (8.4%) and salaries of central-level supervisors (25.6%); whereas during rollout, central-level supervision and transport accounted for 38.1% and 18.6% of total costs, respectively. The higher proportion of costs devoted to transport during rollout is partly explained by the distance from Lusaka and the vast distances between rollout facilities which necessitated two vehicles and two supervisory teams from the central level to conduct supervision visits. Devolution of supervision and quality monitoring systems to district level (as intended by the MOH) should reduce duration of supervisory visits and the associated salary, vehicle and transport costs from Lusaka. As noted, the QA/QC implementation was substantially changed in the transition from pilot to rollout for perceived cost-saving reasons but was carried out inconsistently, if at all; these costs were therefore excluded from the cost analysis.

### Quality

To our knowledge, this is the first study to compare RST QA/QC costs in a programmatic and a research setting. A recent modelling study supported the cost-effectiveness of scaling up syphilis screening within existing ANC programs in eight theoretical country models but excluded the costs associated with training, supervision and quality maintenance [21], which can contribute to both testing quality and cost. Larson et al. (2014) demonstrated the cost-effectiveness of the Zambian RST programme after the first year of implementation in one district. They included cost of testing commodities, HCW time and training but did not address costs associated with supervision and quality monitoring [42]. A Tanzanian pilot RST study addressed this issue [23], documenting the small incremental increase in unit costs incurred by adding a comprehensive quality monitoring system. The average cost per woman screened increased from only $1.92 to $2.74 and the average cost per woman treated from $21.40 to $30.57, reflecting our pilot cost findings, where inclusion of supervision and QA/QC costs augmented the average screening unit cost from $1.49 to $3.19 and treatment unit cost from $14.12 to $30.28. The Tanzanian analysis suggested that devolving supervision and monitoring to district level and reducing frequency of external QA/QC could potentially reduce programme costs, in keeping with the Zambian MOH’s implementation approach. However, the impact of this reduced QA/QC model needs evaluating.

### Implementation Challenges

As noted, several aspects of the pilot study implementation were adapted or scaled down in order to scale-up for a national roll out: training, supervision, QA/QC, supply chain and diagnostic algorithms. Our accompanying qualitative study, Ansbro et al., provides details around actual testing activity and HCW behaviour, allowing examination of our costing data through a richer lens [32]. A number of key challenges were identified during this evaluation of the first phase of the national rollout:

First, cascaded training was utilised in both phases. However, pilot HCWs had much greater and more frequent exposure to central-level research staff who provided monthly supervision and remedial training as required. Second, the reduction in supervision frequency from monthly at all pilot locations to quarterly at selected rollout locations reduced average facility supervision cost by about one-third, from approximately $1,000 to $660. While it resulted in cost-savings, the reduction in supervision frequency afforded fewer opportunities to promote testing and treatment, identify gaps in test kit quality, and to identify and remediate poor provider proficiency or non-adherence to guidelines. Supervision and monitoring of HCWs’ performance has been shown to increase their confidence in test performance and accuracy of test results [46]. Third, while the Zambian MOH was progressive in including QA/QC activities in their national RST programme, devolution to district level introduced challenges during the first rollout phase (Table 6) [47]. On evaluation, rollout HCWs rarely performed incoming kit inspection; district laboratories performed external QC on their own samples but had not initiated an external QA/QC system for surrounding facilities. Several factors may have contributed to this: ineffective planning and communication, lack of nominated responsible personnel, lack of dedicated budget and logistics, lack of local expertise in this type of activity which is often undertaken by NGOs, or lack of hands-on training for district laboratory staff. Since external QA/QC and confirmatory testing were not performed, it is difficult to comment on the quality of testing provided in this preliminary phase of the rollout.

**Table 6:** Changes in implementation methods from NGO-led RST pilot to MOH-led national RST rollout in Zambia.

	PILOT PHASE: 2008–2011	ROLLOUT PHASE: 2012 to present
**Training Model**	Cascaded training: Central workshop conducted by EGPAF/CIDRZ; attendees then provided on-the-job training to facility colleagues.	Cascaded training: District-level workshops conducted by MOH/EGPAF; attendees then provided on-the-job training to facility colleagues.
**Treatment algorithm**	Zambian syphilis treatment guideline pre-RST adoption was three weekly doses of Benzathine Penicillin (BP). During the pilot, patients were given one documented dose of BP following a positive RST test.	Treat with one dose of BP following positive RST result; run RPR confirmation and if active infection confirmed, treat with two additional doses of BP. If RPR confirmation unavailable, continue 2^nd^ and 3rd weekly dose of BP.
**Outcome of integration**	The same HCW offered same-day testing, results and treatment.	RST was variably integrated into patient flow depending on facility-level, HCW cadre and laboratory capacity.
**Internal QC**	In-built control panel	In-built control panel
**External QC**	1) Weekly validation of RST kits with positive and negative control samples; 2) repeat confirmatory testing at a central laboratory of samples collected during study supervision visits.	Validation of RST kits with positive and negative control samples weekly and if a new shipment, new lot number or adverse environmental conditions occurred. Rarely implemented during early rollout phase evaluated here. Knowledge on QA/QC practices was rarely transferred during cascaded training. Control samples were not included in test kits or delivered to facilities by district laboratory personnel.
**Quality Assurance**	Health workers’ accuracy was checked using proficiency panels (sample RSTs prepared with dried tube specimens of serum known to be positive or negative for syphilis) during supervisory visits	Health workers’ accuracy was intended to be checked using proficiency panels sent to the facility by the district laboratory. Not implemented during the early rollout phase evaluated here due to lack of HCW time, lack of dedicated budget, logistics and manpower for QA/QC, inexperience and/or lack of initiative of the district laboratory personnel and lack of on-site lab-training in advance of rollout.
**QA/QC Logistics**	Control samples prepared by research laboratory and transported by study staff to facilities	Control samples were intended to be prepared by district laboratories and transported to the facilities with results transported back to the district laboratory. Not implemented during the early rollout phase examined here.
**Supervision**	Monthly visits from EGPAF/CIDRZ incorporating QA/QC & remedial training for new or poor performing HCWs	Quarterly visits from MOH and EGPAF staff
**Cost Implications**	QA/QC activities contributed significantly to pilot costs, driven by central-level personnel supervision and transport costs	QA/QC rollout costs were reduced due to decentralisation of supervision and quality monitoring to the district level; costs were driven up by higher RST kit cost during rollout and reduced economies of scale due to reduced RST uptake

Fourth, introducing a new POC test with new diagnostic algorithms posed potential challenges and cost implications during the rollout. Our qualitative data shows that several facilities did not adhere to guideline diagnostic algorithms [32]. In sites without confirmatory RPR testing, inadequate HCW training and QA/QC systems may have contributed to inaccurate test result interpretation and unnecessary and costly repetition of RST. If DH1, a high ANC volume site with RPR capability and relatively high RST reactivity rate (7.1%), provided 100% testing coverage as per guidelines, large numbers of positive RST tests would require RPR confirmation; performing a second test incurs higher costs; and RPR-capable sites are still vulnerable to the pre-existing barriers to RPR testing, including supply chain weaknesses, absent or inadequately trained staff and patient loss-to-follow-up. The most cost-effective and usable testing algorithms for areas of varying syphilis prevalence have yet to be established.

Finally, RST supply was consistent during the pilot but less so during rollout. Pilot kits were delivered by study staff during supervisory visits. During the preliminary rollout, RSTs were delivered via the national pharmacy supply chain rather than the usual medical supply route; three of the five rollout sites evaluated (RHC3, RHC4, and RHC5) reported RST stock outs during the entire month preceding data collection.

### Study Limitations

Rollout data were collected within five months of the rollout commencing and therefore reflect early implementation issues which may have been resolved in the interim. Our analysis was cross-sectional and therefore limited to the number of people screened and treated; unfortunately, we were unable to assess birth outcomes, including congenital syphilis, to assess programme impact and cost-effectiveness. We could not adequately quantify the additional costs for RPR confirmatory testing during the rollout, because sites were not selected according to diagnostic algorithm but rather by convenience sampling—therefore, costing sites included a variety of facility level laboratory and staffing capacity which employed different diagnostic algorithms. An assessment of the differential in costs between facilities with and without RPR confirmatory testing and with different local syphilis prevalence was not adequately captured by this study but is an area for future research. The cost methodologies were not completely replicated between pilot and rollout as RST stock outs and ANC scheduling prevented direct observation of RST performance during the rollout phase site visits. In addition, we compared pilot and rollout districts with different characteristics including: population densities, syphilis prevalence, and distance from the district health office—which reduces comparability of data between pilot and rollout phases, but reflects the district variations that exist within the country and is an important consideration in scaling up an intervention. Finally, secular trends during the two-year time gap between pilot and rollout data collection could not be accounted for in this analysis; rollout RST data were not available at the original pilot facilities during 2012.

### Further Research

This study demonstrates the potential for further research pairing in-depth qualitative methods with cost analyses to better understand the challenges of POC test scale-up. Recent commentary has called for increased operations research into scale-up to identify the optimum mix of QA/QC which will be cost-effective yet maintain POC test reliability [28] and research to explore the cost-effectiveness of cascaded training. Dual antibody/antigen POC syphilis screening tests currently under investigation may address issues around the RST antibody test’s lack of specificity for active infection, which inevitably leads to over-diagnosis and treatment of pregnant women [48]. Dual HIV and syphilis antibody tests may potentially contribute to economies of scale in terms of start-up, training, QA/QC and supervision and monitoring, while also serving to increase syphilis screening to match existing high HIV testing coverage in ANC settings [49–51]; further implementation research on the cost and feasibility of dual test deployment is needed. In addition, the WHO is currently investigating the impact and cost-effectiveness of varying syphilis diagnostic algorithms using combinations of RST, dual tests and RPR, according to local prevalence. The results of these studies are awaited.

## Conclusion

This study explored the cost of integrating RST into ANC clinics in both pilot study and national rollout settings in Zambia, reflecting findings from studies performed in other low- and middle-income countries. Cost differentials between pilot and rollout, including the significant influence of syphilis prevalence on unit cost per person screened and treated, and the considerably lower costs of supervision and QA/QC systems, were influenced by an increase in RST kit price, lower testing coverage and by challenges in implementation of training, regularity of testing, supervision, and QA/QC programme components. Planning for further scale-up of the Zambian RST programme must take heed of the ongoing budgetary, supervisory and policy-level support that is required for successful implementation.

There is a growing consensus that despite the additional cost, robust training, QA/QC and supervision systems are essential to ensuring the reliability and reproducibility of POC tests [15,52,53]. We recommend that these implementation programme aspects are included in future models of the cost-effectiveness of RST testing programs. The transfer of responsibility for QA/QC to district-level staff may require greater emphasis in training, specific budget allocation and prolonged supervision and monitoring until they are well established. Involving district-level personnel in supervision could increase opportunities for external QA/QC and remedial training at facility-level. Integrating transport of QA/QC samples and RST kits within existing supply chain mechanisms could potentially improve quality and consistency of testing at little added cost. Given these findings, continued RST scale-up efforts in Zambia could benefit from quality spot-checks to identify remedial support needs for training and QA/QC activities.

## Supporting Information

S1 Table Quality Control and Quality Assurance Associated Costs. (XLSX)

S2 Table Input Data and Key Assumptions. (XLSX)
